# Epithelial Barrier in the Nasal Mucosa, Related Risk Factors and Diseases

**DOI:** 10.1159/000528969

**Published:** 2023-02-01

**Authors:** Rong Zhang, Lan Zhang, Peishan Li, Kaiyun Pang, Huixia Liu, Li Tian

**Affiliations:** ^a^College of Clinical Medicine, Chengdu University of Traditional Chinese Medicine, Chengdu, China; ^b^Department of Otorhinolaryngology, The Teaching Hospital of Chengdu University of Traditional Chinese Medicine, Chengdu, China

**Keywords:** Epithelial barrier, Allergic rhinitis, Chronic rhinosinusitis, Physical barrier, Tight junctions, Chemical barrier, Risk factors

## Abstract

As the first line of defense against risk factors, the nasal epithelial barrier maintains homeostasis in nasal mucosa. The composition of the epithelial barrier contains physical, chemical, immune, and microbiological barriers. Together, these barriers form the nasal defense against irritations. Risk factors from both internal and external environments can disrupt them. External risk factors contain allergens containing proteases, bacteria, virus, particulate matter, diesel exhaust particles, and cigarette smoke. In the meantime, inflammatory cytokines also increase the load on the barrier. Taking into account the role of the epithelial barrier in the nasal mucosa, some studies focus on the treatment of allergic rhinitis (AR) and chronic rhinosinusitis (CRS) by restoring the epithelial barrier, and some progress has been made. Among the therapeutic approaches, histone deacetylase (HDAC) inhibitor and steroid corticosteroids are considered two of the more studied categories, and their roles in repairing barriers have been demonstrated in AR and CRS. The underlying mechanism of HDAC inhibitor may be related to the transcription factor p63. And the protection of corticosteroids may be associated with the allergic disease susceptibility gene, protocadherin-1. Notably, manipulation of the microbiological barrier also has a positive effect on AR and CRS. *Lactococcus* and probiotics are two categories that are worth being explored continuously. We here review and discuss the compositions and risk factors of the nasal epithelial barrier. Furthermore, some novel and promising approaches to restore the defective barrier in nasal allergic diseases were mentioned.

## Introduction

Epithelial barrier is the first line of defense against risk factors in nasal diseases, like allergic rhinitis (AR) and chronic rhinosinusitis (CRS) [1]. It helps to maintain functions and epithelial homeostasis in the nasal mucosa [2]. The dynamic change of the epithelial barrier covers almost all pathological progress during the nasal immune responses. When epithelial cells recognize foreign substances trapped in the nasal mucosa, barrier disruption occurs in successions. Challenges are posed to the integrity and function of the epithelial barrier [3]. Dysfunction of epithelial barrier allows the risk factors to infiltrate downward and induce immune responses in the nasal mucosa. These responses contain permeability increasing, excessive mucus producing, sneezing, and itching triggering, etc. During this immune response, some inflammatory cytokines are produced as well, further disrupting the epithelial barrier and exacerbating inflammation. Therefore, the integrity and function of the nasal epithelial barrier are inextricably bound up with the pathology of allergic diseases. In 2017, Pothoven and Schleimer proposed the barrier hypothesis to better explain the development of allergic diseases [4].

The epithelial barrier physically separates the environment from the body to defend against external exposure. Its dysfunction makes it possible to induce some nasal diseases [5], particularly allergic diseases. Recent studies reported that the epithelial barrier would be impaired by a considerable number of risk factors. The factors, including protease-containing allergens, bacteria, virus, particulate matter (PM), diesel exhaust particle (DEP), and cigarette smoke (CS) [6, 7, 8, 9], can compromise the integrity and impact the function of the nasal epithelial barrier. When the epithelial barrier is disrupted, these factors infiltrate the submucosal space, thus leading to a cascade of immune responses in the nasal mucosa. Epithelial barriers are mainly divided into physical, chemical, immune, and microbiological barriers. The physical barrier of the nasal epithelium divides the internal and external environment and is important to protect against allergens, pathogens, and other irritants. It refers to tight junctions (TJs), adherens junctions (AJs), desmosome, and other compositions. The chemical barrier, including mucus and cilia, traps the inhaled allergens, pathogens, etc., and prevents their invasion. Immunoglobulins (Igs), antimicrobial proteins, and peptides form the immune barrier and thus counteract the immune response on the mucosa. Finally, the microbiological barrier is the microbiota that colonizes the nasal mucosa, and they have also been reported to defend against external stimuli and modulate immunity.

These diseases associated with epithelial barrier disruption in nasal mucosa mainly include AR and CRS [10, 11]. Current epidemiology showed that the prevalence of AR and CRS increased during the past centuries [12, 13]. It is reported that the prevalence of AR in major metropolitan cities in China rose from 11.1% to 17.6% between 2005 and 2011 [14]. Besides, the International Study of Asthma and Allergy in Childhood (ISAAC) found a slight increase in the prevalence of AR in children worldwide, suggesting that AR has a trend of getting younger [15]. As a representative disease of nasal allergy, AR is a chronic noninfectious inflammatory disease and essentially a hypersensitivity reaction mediated by IgE. It is primarily induced by risk factors in the external environment. The epithelial barrier will be disrupted by allergens containing proteases. Naïve T cells are activated by upstream signaling and differentiated into T-helper 2 (Th2) cells, resulting in the release of Th2 cytokines (interleukin [IL]-4, -5, and -13) and the production of allergen-specific IgE. After exposure to the same allergens again, plenty of allergic mediators (histamine, prostaglandin, etc.) are produced to induce a range of nasal allergic symptoms, such as sneezing, nasal itching, and watery nose [16]. Recently, it was found that AR patients tend to develop sinusitis [17, 18, 19]. A wealth of studies have confirmed the potential relationship between AR and sinusitis [20, 21]. Pathological evidence shows that CRS with nasal polyps (CRSwNPs) represents Th2 reaction and eosinophil (EOS) infiltration [22, 23]. The same epithelial barrier dysfunction has also been observed in CRS [24].

**Fig. 1:**
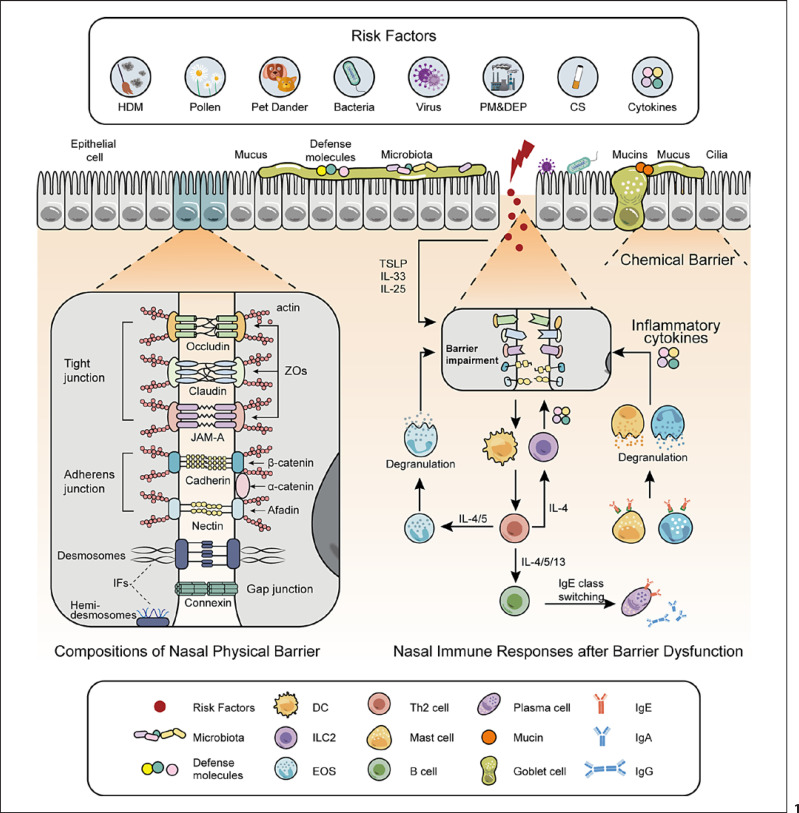
Compositions of the nasal epithelial barrier, risk factors, and immune responses. Epithelial barrier consists of physical, chemical, immune, and microbiological barriers. The physical barrier is mainly composed of TJs and AJs. TJs contain OCLN, CLDNs, JAMs, and ZO. AJs include cadherin/catenin and nectin/afadin complexes. Mucus and cilia form the chemical barrier. Igs and defense molecules (AMPs and proteins) act as the immune barrier. The microbiological barrier refers to the microbiota that colonizes the nasal mucosa. When the epithelial barrier is disrupted by the risk factors, a series of immune responses occur. Differentiated Th2 cells release some inflammatory cytokines and lead to the production of allergen-specific IgE. EOS, mast cells, and basophils are activated and degranulated. The released cytokines and allergic mediators cause continuous damage to the barrier. CS, cigarette smoke; DC, dendritic cell; EOS, eosinophil; HDM, house dust mite; IFs, intermediate filaments; Ig, immunoglobulin; ILC2, group 2 innate lymphoid cell; JAMs, junction adhesion molecules; PM and DEP, particulate matter and diesel exhaust particle; TSLP, thymic stromal lymphopoietin; ZO, zonula occludens.

These findings indicate that the epithelial barrier plays a significant role in nasal diseases. Thus, a hypothesis was proposed that restoring the epithelial barrier could exert benefits for AR and CRS. Some studies focused on restoring the nasal epithelial barrier have been published. Therefore, this review describes the composition and related risk factors of the nasal epithelial barrier as shown in Figure 1. The research progress on promising therapeutic approaches for restoring the defective barrier and their potential mechanisms in AR and CRS are also introduced.

## Compositions of Epithelial Barrier

### Physical Barrier

#### Tight Junctions

The physical barrier mainly comprises cell junctions, including TJs, AJs, and other compositions. The junctions trap and cleanse environmental risk factors. TJs are of crucial importance to maintain the integrity of the epithelium [25]. As the multiprotein complex is located on the apical side of epithelial cells [26] and is one determinant of intercellular permeability, it consists of transmembrane proteins, membrane-associated proteins, and soluble cytoplasmic proteins. They are also in charge of immune surveillance and can prevent the invasion of foreign particles into subepithelial layers [27]. Recent studies suggested that TJs play an unneglectable role in the molecular mechanisms of cell proliferation and differentiation [28, 29, 30]. The transmembrane proteins of TJs are divided into three main groups, including the single-transmembrane domain proteins like junction adhesion molecules (JAMs), the triple-transmembrane domain protein like blood vessel epicardial substance (BVES), and the four-transmembrane domain proteins like claudins (CLDNs) and tight junction-associated Marvel proteins (TAMPs).

Occludin (OCLN) is the first integral membrane protein identified at TJs in 1993 and the reliable immunohistochemical marker for TJs [26, 31, 32], providing a site of attachment for junctional proteins. OCLN is unified with tricellulin and MarvelD3 as TAMPs, tightly linked related Marvel-domain proteins. They have the same tetraspan protein with cytoplasmic N- and C-termini as CLDNs, but their cytoplasmic domains and extracellular loops are different in length and structure. TAMPs are the unique and redox-sensitive markers of TJs and possess the ability to maintain the polarity, regulate the adhesion, and receive the survival signals of cells. Therefore, they could play a role in the epithelial barrier. They interact with zonula occludens (ZO)-1 and regulate cell permeability [31, 33]. Another study investigated that OCLN and tricellulin regulated the complexity of TJ strands to strengthen the epithelial barrier [34]. In human nasal epithelial cells (HNECs), the expression of OCLN was detected and its presence was observed in cell borders [35, 36]. However, some studies have also indicated that OCLN does not play a critical role in TJ and the function of the epithelial barrier. Studies reported that mature TJ strands were still observed in the epithelial cells of OCLN-knockout mice [37, 38]. In recent years, both decreased OCLN protein and mRNA were detected in the nasal epithelium of AR patients compared to controls [39]. Immunofluorescence imaging of pathological specimens revealed that OCLN in specimens from AR patients exhibited disrupted layers and irregular expression patterns [40]. It means that OCLN serves some function in the epithelial barrier, rather than being a necessary structural component in TJs.

Identified in 1998 [41], CLDNs are another main structural component of TJs, whose discovery paved the way for the subsequent discovery of TJs. They basically consist of an intracellular NH2 terminus, a longer intracellular COOH terminus, one short intracellular loop, and two extracellular loops. At least 27 CLDNs have already been identified [26, 42]. They are expressed in all known epithelial tissues. CLDN-1, -4, -7, -8, -12, -13, and -14 had been reported to be detected in the human nasal mucosa [1]. Various CLDNs would show different characteristics. They form both intercellular barriers and intercellular pores. For example, CLDN-1, -3, -5, -11, -14, and -19 are characterized as sealing, whereas CLDN-2, -10, -15, and -17 are characterized as channel forming [43, 44, 45]. Therefore, they play an important role in the regulation of epithelial cell permeability. Transfection experiments of nonpolarized cells revealed that CLDNs were able to form TJ strands in intercellular contacts [46, 47, 48]. Also, electrophysiological studies showed a direct correlation between epithelial resistance and TJ strands. CLDNs build a paracellular channel that can transport ions and small molecules [49]. These channels are permselective for cations, anions, or water. It means that CLDNs and their trans-combinations influence the permeability of TJs for ions and solutes [43, 50]. Among all the detected CLDNs, CLDN-2 is currently the most widely studied channel-forming one. It induces an increase in paracellular permeability to organic cations but does not affect permeability to anions and uncharged molecules [51, 52]. Acting as a barrier or forming pores, CLDNs are capable of charge selectivity in paracellular conductance. In conclusion, CLDNs play an irreplaceable role in the regulation of cell permeability.

Moreover, other transmembrane protein cannot be neglected, like BVES. BVES, a triple-transmembrane protein, is widely observed in the cell junction [53, 54]. Some reports revealed that the disruption of BVES would result in the decrease of cell adhesion with impaired AJ and TJ formation. Ig-like groups of single-transmembrane adhesion proteins act in TJs, like JAMs. JAMs possess a single-transmembrane domain and a postsynaptic density zone (PDZ) domain-binding motif. It can interact with ZO-1, the adaptor protein connecting transmembrane proteins and recruiting other cytoplasmic components. They are mainly expressed at the junction of epithelial cells, which have been detected in primary cells for HNECs [55]. The major types of JAMs have been discovered, including JAM-A, -B, and -C, which share the same short intracellular domain. Concerning their function in the epithelial barrier, evidence has proved that JAMs are necessary for the assembly of TJs [56]. The expression and dimerization of JAMs regulate the barrier function of epithelial cells, which has been observed to increase permeability [57]. It was reported that the disruption of JAM-A dimerization may hinder the formation of scaffolding protein complexes, which would prevent integrin and reduce cell migration [58].

In addition to the aforesaid proteins, adaptor proteins are involved in supporting TJ structures, which are also called peripheral scaffolding proteins. Containing ZO proteins, afadins, membrane-associated guanylate kinases (MAGIs), multi-PDZ domain protein-1 (MUPP1), etc. [59, 60], these peripheral scaffolding proteins interact with the before-mentioned transmembrane proteins but also each other. They formulate the protein network in the structure of TJs. With different and particular C-terminals, ZO-1 and other ZO proteins share the same domain and hence exert a series of interactions via similar structural domains and different terminals [61, 62]. Beyond that, MAGI also possesses the same structural domain as ZO proteins. Unlike the above structure, the domain of MUPP1 is either in an inverted state or has a variety of PDZ domains. More than that, another class of adaptor proteins like junction-associated coiled-coil protein also interact with transmembrane and peripheral scaffolding proteins to recruit to affect signals [63]. These proteins, protein kinases, and other substances play a critical role in subsequent physiological or pathological processes by regulating signaling pathways [29].

#### Adherens Junctions

AJ is a form of cell-cell adhesion structure. AJs are mainly responsible for intercellular adhesion by connecting the cell membrane and skeleton. The functions of AJs are connected with the organization and movement of epithelial cells, internal signaling pathways, and transcriptional regulations [64]. AJs are constituted of cadherins, catenins, nectins, and afadins. They form the structure of AJs and maintain their functions. On the cell adhesion of epithelial cells, cadherins, the first found AJ protein, mediate the Ca^2+^-dependent adhesion [65]. E-cadherin is the classical protein with five repeat extracellular domains also known as extracellular cadherin domains. It can mediate cell adhesion with adjacent cells and participate in interactions to bind skeleton proteins [66, 67]. Catenins are other proteins that can interact with the cytoplasmic structure of E-cadherin. They include α-catenin, β-catenin, and p120-catenin, which construct a complex with cadherin. Synergistically connecting the cytoskeleton and stabilizing the population of actin fibrils [68, 69, 70], the cadherin/catenin complex was also found to be closely linked with ZO-1 in TJs [71]. Nectin, which belongs to the IgG proteins, is the dependent adhesion molecule widely distributed in epithelial cells. The compositions of nectins contain three IgG-like domains [72]. Its cytoplasmic domain directly binds to the acting-binding protein (afadin) [73]. Afadins are actin-binding proteins anchoring to the cytoskeleton and then constituting the nectin/afadin complex. The interactions can directly influence whether cadherin can be localized to intercellular junctions precisely [74].

#### Desmosomes

Desmosomes cannot be ignored in maintaining intercellular adhesion and cellular integrity among the various components of cell junctions [75, 76]. They are primarily responsible for the mechanical stability between adjacent cells and facilitate intercellular communication through signaling transfer. Bizzozero, an Italian pathologist, first observed the structure of desmosomes and revealed their complex structures and organization through subsequent techniques such as electron microscopy. Desmosomes are cadherin-based multiprotein complexes. It consists of three main components, including the desmosomal cadherins, the armadillo proteins, and plakin proteins. They are arrayed on the cytoplasmic surface of the adjacent cell membranes [77]. Desmosomal cadherins consist of desmosomal cadherins termed desmogleins (DSGs) and desmocollins. They are transmembrane proteins whose extracellular domains form the adhesion interface of desmosomes. Their cytoplasmic tails anchored to the desmosomal plaques [76]. The desmosomal cadherins can bind CA^2+^ and present a rigid functional conformation, thus strengthening adhesion between adjacent cells [78]. A study found that the expression of DSG2 and DSG3 in human nasal polyps was significantly reduced compared to their expression in the normal nasal mucosa. In vitro experiment revealed that exposure to Th1 and Th2 cytokines resulted in reduced expression and cleavage of DSG2 [79]. The armadillo proteins comprise plakoglobin (PG) and plakophlins. PG, also known as γ-catenin, is an adaptor protein that involves in promoting the adhesion of intermediate filaments, regulating the compositions of desmosomes and mediating signal transduction. PG and β-catenin are highly homologous. It is capable of translocating to AJs and binding E-cadherin. Since plakophlins show a similar tissue- and differentiation-specific pattern like desmosomal cadherins, it also interacts with desmoplakin and DSG [80, 81, 82]. Plakin proteins, another component of desmosomes, interconnect and organize the intermediate filaments, microtubules, and microfilaments of the cytoskeleton and anchor them to membrane-associated structures. Seven members of the plakin proteins have been identified, including plectin, bullous pemphigoid antigen 1, desmoplakin, microtubule and actin cross-linking factor 1, envoplakin, periplakin (PPL), and epiplakin [83, 84]. PPL was found to act in airway epithelial injury. Nasal polyps are also pathologically characterized by the alterations of airway epithelial cells (AECs), such as dysfunction of epithelial barrier and deficiency of desmosomes [85]. Thus, they suggest that PPL may be crucial in nasal polyposis. Desmosomes act a part in cellular integrity due to their wide distribution, complex structure, and multiple functions. The disruption of their composition and structure affects cell adhesion.

#### Gap Junctions

Gap junctions comprise an array of intracellular channels formed by connexin (Cx) proteins and are actively involved in intracellular communication between adjacent cells, which can transport ions and small molecules (<∼1.2 kDa) [86]. Cxs are made up of four membrane-spanning structural domains, two extracellular domains, as well as one cytoplasmic C-terminus necessary for intracellular communication [87]. Most Cxs have already been identified in the sinus mucosa [86, 88, 89]. Cx43, as the most classic Cx, has been reported to be detected in human epithelial cells isolated from nasal polyps [90].

### Chemical Barrier

#### Mucus and Cilia

Mucus is the frontline exposed to risk factors in the nose. The mucus can clean the mucosal surface by flushing irritants and forms a mucosal protective layer. Its cleaning ability refers to the nasal mucociliary clearance (MCC), which is a prominent physiological and pathological feature of nasal disease. Mucus and cilia are the two main and complexed parts in the MCC. Impairment of either of them can lead to abnormal nasal secretions and persistent accumulation of local irritation [91]. Mucus is a dynamic translucent barrier that can exchange molecules, transfer, and remove foreign particles, which has the significant functions of clearing foreign particles and participating in innate immune defense. Reportedly, the airway needs to remove and transport 25 million particles per hour [92, 93, 94]. Mucus heterogeneously mixes multiple components from goblet cells and submucosal glands, including secreted peptides, cells, cellular debris, proteins, water, salts, enzymes, and bacterial products. Among the composition of mucus, other substances take part in preventing the invasion into the epithelium, such as IgA, albumins, and antimicrobial peptides (AMPs) [95]. Mucins (MUCs) secreted by epithelial cells and goblet cells are one of the significant macromolecular components in airway mucus [96]. Existing research confirmed that MUCs have nine classes in the human airway mucosa: MUC1, MUC2, MUC4, MUC5AC, MUC5B, MUC7, MUC8, MUC11, and MUC11 [93]. MUC5AC and MUC5B with high molecular weight provide the biophysical properties for mucus transportability [97]. As a bacterial adhesion receptor analog, MUCs confine these bacteria through binding to bacteria and thus assist in clearing irritants on the surface of the epithelium [98]. Moreover, MUCs are also involved in regulating the rheological and physical properties of mucus [99]. Cilia are an essential cellular structure of the airway mucosa. Normal cilia are cylindrical protrusions from the apical surface of epithelial cells and are anchored by intracellular basal bodies [100]. Cilia beat in a coordinated manner and convey mucus to various drainage sites, thus removing continuous stimulated allergens, pathogens, bacteria, viruses, etc. [101, 102]. Under pathological conditions, the normal structure and function of the cilia are altered. Thus, the nasal mucosal ciliary clearance is weakened [99].

### Immune Barrier

#### Immunoglobulins

As a result of the immune response to antigen stimulation, the immune barrier of the nasal mucosa protects the organism from damage. It is composed of Igs and lymph nodes. Igs, mainly IgA and IgG, serve as important members of the immune barrier of the nasal mucosa [103]. They play an important role in nasal allergic diseases. They are secreted by plasma cells distributed in the lamina propria of the nasal cavity. After T cells and B cells are activated by external antigens, the immune system triggered by plasma cells produces secretory IgA and IgG-based immunological effector molecules. As an important effector molecule of mucosa immunity, IgA plays a role in immune clearance, regulating microecological balance, inducing immune tolerance, and suppressing inflammation and allergic reactions [104]. It is involved in the frontline defense mechanisms of the respiratory tract. Through the formation of a protective layer on the mucosa, secretory IgA removes antigens more effectively together with the mucus [105]. As one of the important antibodies in humoral immunity has the function of phagocytosis, agglutination, precipitation of antigens, and neutralization of toxins, it performs immune clearance functions by recruiting natural immune cells with phagocytic properties. Generally, lymph node tissue also acts as an immune barrier. Lymph node tissue in the nose refers to nasopharyngeal-associated lymphoid tissue (NALT). In the human nasal mucosa, especially in the middle turbinate of children, NALT-like structures of follicle-forming lymphocyte aggregates have been found [106]. It means that NALT contains the lymphocytes required to induce and regulate mucosal immune responses against antigens delivered to the nasal cavity [107]. However, it is worth noting that the location of NALT does not fall under the category of nasal cavity and sinuses; thus, the role it plays in the immune barrier in nasal diseases (e.g., AR and CRS) needs to be confirmed.

#### Antimicrobial Proteins and Peptides

In addition to mucus on the surface of the nasal mucosa, a number of host defense molecules play an important role, including various antimicrobial proteins and AMPs. They serve as the components of the innate host defense of the mucosa. Their functions can be divided into two main categories, which are the antibacterial effects and the immunomodulatory activities. They modulate mucosal inflammation, chemotaxis, antimicrobial defense, antioxidant levels, and repair remodeling. The major antimicrobial proteins include lysozyme (LYZ), lactoferrin (LTF), S-100 protein, etc. [108]. Below is presented the description of each of these antimicrobial proteins. LYZ and LTF are the most abundant antimicrobial proteins in the airways [109, 110]. They can be produced by AECs, neutrophils, and macrophages [111]. In the early histochemical analysis of turbinate tissue, it is indicated that LYZ and LTF are present in the plasma cells of the submucosal glands [112]. The report proposes that LYZ hydrolyzes bacterial cell walls and modifies mucus viscoelasticity in the nasal epithelium [113, 114]. LTF acts as an iron chelator and collaborates with LYZ to combat bacterial growth [115, 116]. In one study reported, increased immunoreactivity of LYZ in mucosal biopsy specimens in cases of CRS was observed [117]. Relative to the AR alone and control groups, decreased levels of LYZ and LTF were found in the nasal secretions of patients with AR with CRS [118, 119]. In addition, lung and I-nasal epithelium clone proteins are another group of antimicrobial proteins secreted by AECs. It has been reported that short lung and I-nasal epithelium clone proteins expression is reduced in eosinophilic CRSwNP, compared to noneosinophilic NP, and its mRNA level expression is selectively inhibited by IL-4 and IL-13. Moreover, the S100 protein is a family of low-molecular weight proteins that also possess direct antibacterial effects. Isoforms of S100 have been shown to play important roles as alarms, AMPs, etc. [120]. Both S100A8 and S100A9 were reportedly detected in the nasal lavage fluid of AR patients [121]. S100A7 was reported to be reduced in nasal secretions from AR, while S100A7, S100A8/S100A9 were reduced in CRS with or without NP [122]. Also known as host defense peptides, AMPs are considered the rapid and first-line response of the innate immune system. By inhibiting bacterial proliferation, capturing and killing pathogens, AMPs play an important role in innate host defense [123]. Many AMPs exhibit antibacterial activity against Gram-positive and -negative microorganisms, fungi, unicellular protozoa, and viruses [124, 125, 126]. In addition, AMPs not only induce cell migration, proliferation, and differentiation but also regulate the production of cytokines/chemokines [127]. Defensin and cathelicidin (LL-37/hCAP-18) are the most well-studied AMPs in humans. Defensins are widely distributed polypeptides with three-dimensional folding characteristics [128]. It kills bacterial and fungal peptides and induces and enhances appropriate adaptive responses [129]. Defensins are subdivided into α- and β-defensins. To be specific, it is reported that α-defensin can enhance lung epithelial cell proliferation to promote epithelial repair [130]. In the nasal mucosa, human β-defensin 2 is induced by bacterial lysates to defend against infection [131]. Expressed in human respiratory epithelial cells, mast cells, and other cells, defensins have demonstrated antibacterial activity against bacteria, viruses, and fungi. Meanwhile, the study reported that LL-37 also played an anti-inflammatory role in the lipopolysaccharide-induced inflammation model [132, 133]. Compared to control groups, there was a reduced LL-37 level in children with AR [134]. There was a negative correlation between LL-37 levels and disease severity. Taken together, these defense molecules in the nasal mucosa are part of the chemical barrier in the nasal epithelium. They are also shown to be associated with nasal diseases. However, it is noted that studies on antimicrobial molecules in the nasal mucosa are still scarce and at low levels.

### Microbiological Barrier

#### Microbiota

The hygiene hypothesis, which is a theoretical assumption proposed by Strachan in 1989, links the occurrence of allergies and some other autoimmune diseases with deficiencies in hygiene practices [135, 136]. Recently, further development has been achieved in the hygiene hypothesis whose focus has gradually shifted to the influence of microbiota in these aforesaid diseases [137]. Its development contains microbiota coexisting with the human body and its abundance and diversity are involved in immune homeostasis [138]. In general, microbiota resides in the mucus of the mucosal surface, including gastrointestinal and airway tracts. The nasal mucosa, as a part of the upper airway, is also colonized by microbiota. Prior studies have revealed that microbiota diversity has a relationship with the sensitization of allergens [139]. For this reason, microbiota gets involved in the physiology and pathology of some allergic diseases in the upper airway. Microbiota is usually located on the surface of the epithelial cells and in the mucus. When the microbiota move beneath the epithelium, the immune system is stimulated, and the inflammatory process is promoted [5]. The roles of microbiota in airway mucosal diseases include the influence of early exposure to a rich-microbiota environment on susceptibility to diseases before adulthood and the influence of microbiota on physiological processes in the immune system [140]. Microbiota residing in the nasal mucus is usually grown in infancy and continuously remodeled by environmental exposure at a later stage [141, 142]. Advances in molecules and bioinformatics have greatly expanded the understanding of microbial compositions and functions. It was shown that microbiota which is early exposed to the external environment might exert an influence on the immunity to Th2 immune reactions [143]. Compared with healthy infants, those susceptible to allergy were found to have a greater abundance of Oxalobacteraceae and Aerococcaceae families [139, 144, 145, 146]. It has been proved in vivo that a drop occurs in the susceptibility to allergens in neonatal mice which have induced allergic inflammation after the development of the airway microbiota [147]. With the increase of age, the early colonizing microbiota mentioned before is replaced by Streptococcaceae, Corynebacteriaceae, or Moraxellaceae [148]. *Staphylococcus aureus* (*S. aureus*), *Propionibacterium*, *Prevotella*, *Corynebacterium*, *Bacteroides*, and *Streptococcus* are the significant strains of the nasal mucosa. Some reports showed that the diversity of the nasal microbiota in nasal diseases presented both increasing and decreasing trends [149, 150]. Also, research suggested that major nasal microbiotas are similar, whose abundance and diversity however differ. Chen et al. [149] claimed that the decreased diversity of the nasal microbiota is detected in AR. Lal et al. [151] reported that the abundance of *S. aureus*, *Propionibacterium*, *Corynebacterium*, and *Peptoniphilus* increased, whereas that of *Prevotella* and *Streptococcus* decreased. Moreover, the group of Gan demonstrated that the abundance of Spirochete, *Pseudomonas*, and Peptostreptococcaceae increased in AR, while that of *Lactobacillus* declined. The abundance of *Moraxella* decreased in CRS and that of *Haemophilus* increased [152]. However, different regions of the nasal cavity show various microbial diversity [153]. For example, the microbiota of the middle meatus was significantly more diverse than that of the anterior nares [154]. One study that sampled the different positions of the nose, including the nasal vestibule, middle meatus, maxillary sinus, ethmoid culture, superior meatus, and sphenoid in CRS patients, discovered the microbial variability in different sites [153]. It is noted that no uniform sampling site has been identified for the nasal microbiological barrier. It needs to be paid attention to in the future.

Meanwhile, a significant interaction exists between the mucosal microbiota and immune cells. Related studies have been mostly carried out in the field of gut microbiota. Both microbiota and its metabolites have effects on immune cells. It has been demonstrated that short-chain fatty acids, one of the metabolites, act as a histone deacetylase (HDAC) inhibitor in immune reactions and are involved in maintaining the integrity of epithelial cells [155, 156]. It is suggested that airway microbiota can also induce similar effects on airway immune reactions given the similarity of intestinal and airway epithelial barriers [157]. Limited studies have recently focused on the impact of the airway microbiota on immune cells in localized allergic reactions. It is proved that the colonization of the inflammatory microbiota induces immune responses in the airway mucosa. The microbiota exacerbates inflammatory responses and activates toll-like receptors in the nasal mucosa [158, 159, 160]. On top of this, it seems that microbiota is involved in the role of innate lymphoid cells (ILCs) in innate immune reactions. Belonging to the heterogeneous family of innate lymphocytes enriched in the mucosal barrier [161], ILCs are regarded as an important component of innate immunity to protect against pathogens. It was noticed that ILC3, another subset of ILCs, limits the expansion of allergen-specific CD^4+^ T cells through a mechanism related to its presentation of microbial antigens in the gastrointestinal tract [162, 163, 164]. Nevertheless, still few studies focus on the hypothesis which remains to be explored and confirmed by conducting more studies.

## Risk Factors Contributing to Epithelial Barrier Disruption

### Allergens Containing Proteases

Environmental exposure is a crucial determinant of nasal diseases like AR and CRS. Among these environmental factors, protease-containing allergens are one of the most dominant. It is known that the nasal epithelial carrier can be undermined by allergens containing proteases, mainly from house dust mites (HDMs), pollen, pet dander, insect, fungi, etc. They can induce immune reactions, degrade barrier proteins, and enhance epithelial permeability through protease-activated receptors. HDM, one of the most critical allergens, produces cysteine and serine proteases to compromise the epithelial barrier [165]. Among various mites, *Dermatophagoides pteronyssinus* (*D. pteronyssinus*) 1 was the main identified allergenic protease, which possesses cysteine and serine protease activities. It was discovered that HDM impairs the physical barrier by cleaving OCLN and CLDN, inducing the intracellular protein hydrolysis of ZO-1, decreasing transepithelial resistance (TER), and increasing fluorescein isothiocyanate-dextran 4 kDa permeability [166, 167, 168, 169]. Pollen is another large group of allergens with protease activity. For example, that pollen from Japanese cedar and cypress as well as Rocky Mountain juniper contains serine and aspartic acid proteases [170, 171]. It is reported that proteases released from pollen disrupt TJs and AJs in the physical barrier, including impairing OCLN, CLDN-1, ZO-1, and E-cadherin [172, 173, 174]. In addition, insects and fungi also contain proteases, which can act as not only the stimulator but also the promoter of allergic reactions and aggravate responses. They can also have a negative effect on the epithelial barrier.

### Bacteria

As has been mentioned above, the microbiological barrier, which is the microbiota, plays a role in the nasal immune responses. The microbiota plays a protective and regulatory role in the mucosal immune system. However, some pathogenic bacteria can also have a damaging effect on the nasal epithelial barrier. It is reported that, by disrupting epithelial barrier function, promoting type 2 inflammation, and driving polyp formation, *Staphylococcus aureus*, which is the primary bacteriological suspect in CRS, modulates innate and adaptive immunity [175]. Unfortunately, it can be found that research on pathogenic bacteria on the nasal epithelial barrier is still at an early stage. In view of this research direction, there are some advances in the intestinal epithelium and airways. *Escherichia coli* (*E. coli*)-stimulated intestinal epithelium shows the downregulation, dephosphorylation, and translocation of TJ protein expression (CLDN-1, ZO-1, -2, and E-cadherin) [176, 177]. In addition to the decrease of TER, *E. coli* disrupts the ultrastructure of the mitochondrial morphology of epithelial cells, as evidenced by the presence of distorted cristae in the intact outer membrane [178]. In the polarized bronchial epithelial cells, *Burkholderia cenocepacia* alters the permeability and migratory capacity [179]. It has been reported that *Pseudomonas* elastase disrupts AECs and hydrolyzes TJ proteins to increase the permeability [180]. It also altered the distribution of TJ proteins (ZO-1 and OCLN) and was able to cross polarized airway epithelial monolayers [181]. *Legionella pneumophila* was found to induce MUC5AC through ERK/JNK and NF-κB pathways [182]. *Vibrio cholerae*, a zinc-containing metalloprotease, was found to degrade OCLN specifically in MDCK-1 cells [183]. It implies that an important part of bacterial pathogenesis may be the specific degradation of important host proteins by bacterial zinc-containing proteases. To sum up, bacterial damage to the epithelial physical and chemical barriers has been partially confirmed by studies, mostly in the airway and intestinal epithelium.

### Virus

Increasing evidence indicates that viral infections both cause impairment to the physical barrier and increase the permeability of the epithelial cells. Human rhinovirus (HRV) infection is one of the most common viral infections in the nasal mucosa. First of all, intranasal attack of HRV weakens the ability of the nasal mucosa to clear [184]. Second, HRV has a disruptive effect on epithelial barrier function. As evidenced by decreased expression of ZO-1, E-cadherin, CLDN-1, and OCLN, HRV incubation induced disruption of barrier proteins in vitro [185]. In the meantime, HRV resulted in increased ROS production, decreased activity of protein phosphatase, and increased protein complexinase phosphorylation levels. In addition, HRV-stimulated HNE experiments also show a reduction in TER. This means that HRV inhibits TJ and AJ proteins, thereby decreasing TER and increasing epithelial barrier permeability to larger molecules [186]. Similar results were uncovered in AECs, where HRV infection decreased TJ expression at the epithelial barrier, reduced epithelial TER, and dissociated ZO-1 from TJ [187, 188, 189]. HRV can disrupt epithelial barrier function, and provide an environment for subsequent allergens to cross the epithelial cell layer and secondary responses [190].

### Particulate Matter and Diesel Exhaust Particles

After the Industrial Revolution in the 19th century, industrialization posed a huge threat to human health on a global scale. Byproducts of industrial development, such as PM2.5, PM10, and DEP, have increased the pressure faced by the nasal mucosa. Numerous studies have confirmed that these substances are in connection with the prevalence of nasal diseases to some degree. It was demonstrated that these particles could undermine the integrity of the physical barrier, including downregulating CLDN-1, ZO-1, and E-cadherin expression [7, 191, 192]. Experiments revealed that PM2.5 decreased TER and fluorescein isothiocyanate-dextran 4 kDa permeability on the nasal mucosa. In addition to the physical barrier, these environmental particles also affect the chemical barrier. The first interaction with these particles is the mucus and cilia [193, 194]. DEP has been reported to induce elevation of MUC5B/MUC16 in human bronchoalveolar lavage fluid (BALF) [195]. PM2.5 induced the MUC5AC/MUC5B gel-forming MUC expression ratio and downregulated the ciliated cell expression program significantly in the nasal epithelium [196]. This leads to abnormal mucus secretion and ciliated cell dysfunction from the nasal epithelium. This evidence suggests that PM and DEP drive both physical and chemical barriers.

### Cigarette Smoke

As one of the proven causes of various airway diseases, CS was reported to affect the epithelial barrier at multiple levels to induce the airway inflammation [197, 198]. First of all, CS exposure would lead to the disrupted physical barriers, including impaired TJs and AJs [199]. Experiments in human sinonasal epithelial cells proved that CS extract reduced the expression of TJ proteins such as OCLN, CLDN-7, ZO-1, ZO-2, and JAM-A [198, 200]. Expression of CLDNs, OCLN, E-cadherin, JAM-A, and ZO-1 has also been observed in the airway epithelium [201]. It also decreased the TER and increased its permeability in a dose-dependent manner. Apart from the physical barrier, CS negatively affects nasal epithelial ciliogenesis, activity, and function, thus affecting the mucosal ciliary clearance apparatus [202, 203]. CS also induced the decrease in airway cilia and the increase in MUCs [204, 205]. Moreover, CS extract was reported to induce low levels of LL-37, which is belonged to the immune barrier and was able to prevent the disruption of OCLN and ZO-1 [201]. Lastly, it is found that CS extract significantly alters the secretion of nasal AMPs, affects the nasal microbiota, and reduces the apical secretion of chemokine ligand 20 [206]. It means CS extract impairs the chemical and immune barriers in the nasal mucosa. The potential mechanism by which CS affects the epithelial barrier of the nasal mucosa is akin to the oxidative stress mechanism by which DEP affects the barrier. In summary, CS compromises the physical, chemical, and immune barriers of the nasal epithelium, thereby stimulating and exacerbating the nasal mucosal immune response.

### Inflammatory Cytokines

In addition to the environmental factors described above, inflammatory cytokines produced during immune reactions can induce and exacerbate damage to the epithelial barrier as well. Th2 cytokines not only enhance the activation of inflammatory cells but also disrupt the epithelial barrier by impairing cell junctions. IL-4, a classical Th2 cytokine, plays an important role in nasal allergic diseases. Reports revealed that IL-4 disrupted the epithelial integrity and reduced ZO-1 and OCLN expression in pulmonary neuroendocrine cells [167]. In vitro experiments on 16 human bronchial epithelial cells also confirmed that IL-4 and IL-13 increased epithelial permeability [55]. They reduced TER, JAM-A, and E-cadherin expression and increased CLDN-2 expression in sinus epithelial cells. Meanwhile, damage to the nasal epithelium is inevitably associated with the occurrence of environmental aggressions. The cytokines produced during the damage and death of epithelial cells are called epithelial cell-derived cytokines, including thymic stromal lymphopoietin (TSLP), IL-25, and IL-33. These cytokines are key regulators that link epithelial mesenchymal communication and induce Th2 reactions in the epithelium [207]. TSLP, an IL-7-like cytokine, is a characteristic role of allergic diseases. It has two isoforms, including long-form TSLP and short-form TSLP. Several studies reported a rise in TSLP in allergic diseases. It was revealed that long-form TSLP acted on the epithelium and disrupted the barrier function of epithelial cells via signal transducer and activator of transcription 5 [208]. However, some studies reported that TSLP can upregulate OCLN, CLDN-1, -4, and -7 to enhance barrier function as well [209]. However, it did not mention the specific isoform of TSLP.

## Approaches for Restoring Epithelial Barrier in Nasal Diseases

The relevance of the nasal epithelial barrier to nasal disease has been well studied. The investigation of therapeutic approaches to protect and restore the epithelial barrier has been prioritized. Avoiding the external triggers is the first step in preventing the disruption of the epithelial barrier. Some novel and promising therapeutic approaches that may restore the disrupted epithelial barrier are also found. They include biologics, steroid corticosteroids, natural products, and microbiota. In this chapter, they will be discussed according to AR and CRS. Their potential mechanisms and the difficulties they faced will also be discussed.

### Allergic Rhinitis

Current research revealed that the dysfunction of the nasal epithelial barrier allowed allergens to permeate into mucosal tissues, ultimately giving rise to mucosal immune reactions. Epithelial barrier is a key segment in the pathogenesis of nasal inflammation and is likely to correlate with the severity of the inflammatory response. It was detected that the proteins and mRNA of TJs and AJs were decreased in AR patients and Der p 1-induced experiments, including OCLN, CLDN-1, -3, -7, -12, ZO-1, and JAM-A [39]. Both a simultaneous decrease in TER and an increase in cell permeability were also observed. With the reduced expression of TJs, the intercellular structure becomes looser and more irregular [40, 210]. Meanwhile, the permeability of the nasal epithelium increases owing to allergen stimulation, which accelerates the transmission of allergens through the nasal epithelial barrier. It is suggested that restoring the epithelial barrier may be a new way to treat AR in consideration of the advances in this field. As a result, a lot of studies have begun to investigate the treatment of nasal diseases by targeting the epithelial barrier. The main relevant therapeutic approaches are composed of HDAC inhibitors, corticosteroids, natural products, etc. In this section, each of these approaches will be described and some possible mechanisms will be mentioned.

HDAC inhibitors are one of the more promising classes of therapeutic measures. Through promoting chromatin condensation and inhibiting gene transcription, it is reported that HDAC can affect the function of the epithelial barrier [211, 212]. A study targeted at the repair of the epithelial barrier noted that the activity of HDAC was inversely correlated with the integrity of the epithelial barrier [213]. It demonstrates a potential link between the barrier function, HDAC activity, and AR pathology [214]. HDAC activity increased chromatin accumulation, delayed cell division, fixed pro-inflammatory factor profile, and delayed healing of chronic inflammation. In primary nasal epithelial cells of AR patients, JNJ-26481585, which is a second-generation HDAC inhibitor, not only increases the integrity in a dose-dependent manner but also reconstructs physical barrier defects by promoting TJ protein expression and reorganizing the TJ network [213]. Also, this result was also confirmed in the bronchial epithelial barrier which has a similar structure to the nasal mucosa. HDAC inhibitors reconstitute defective bronchial epithelial barrier function and stimulate TJ expression, which is involved in attenuating barrier dysfunction, inhibiting airway inflammation, and reducing hyperresponsiveness [215]. However, the effects of HDAC inhibitors toward biological targets are reversible [213]. It indicates that the maintenance of HDAC inhibitors' effects required continuous dosing. Second, steroid corticosteroids are considered the first line of therapy for nasal disorders. To achieve anti-inflammatory effects, it suppresses allergic inflammation by regulating cytokines secreted by immune cells. A recent study has shown that both fluticasone furoate and mometasone furoate significantly restored epithelial barrier integrity and reduced mucosal permeability in both AR patients and mice [216]. As for the effect of corticosteroids on TJ, it is shown that they increased the mRNA and protein levels of TJ like OCLN. Glucocorticoids were reported to significantly influence transepithelial electrical resistance (TEER) and paracellular permeability in the pulmonary epithelial barrier [217]. Its regulation of CLDN-8 and induction of OCLN recruitment in TJ was observed [216]. The latent mechanism to promote epithelial barrier integrity may be related to induction of glucocorticoid to protocadherin-1 (PCDH1) [218, 219]. PCDH1, an airway hyperresponsiveness susceptibility gene, has an important role in the intercellular junctions of the airway epithelial barrier. Only a small number of the discovered susceptibility genes related with allergy disorders, such as PCHD1, have been shown to be associated with the epithelial barrier [220]. It is reported that knockdown of PCDH1 hindered the formation of physical barriers at the apical surface of normal AEC lines, such as TJs and AJs [218]. Glucocorticoids have been revealed to induce the expression of this gene. It has also been shown that dexamethasone induced the production of PCDH1 isoform-2, which is accompanied by an increase in epithelial barrier function [218]. Therefore, corticosteroids promoting epithelial barrier integrity by increasing PCDH1 is a future-worthy method. In the meantime, the functions of individual PCDH1 variants cannot be neglected. Finally, some natural products are capable of producing protective effects against physical barrier defects. A study found that piper nigrum extract ameliorated and reduced OVA-specific antibody levels and histamine released from mast cells, thereby suppressing nasal allergy symptoms [221]. In the intravenous Evans blue injection results, it reduced the elevated permeability and prevented epithelial detachment from the nasal mucosa, for the purpose of protecting epithelial integrity. Western blotting analysis suggested that piper nigrum extract not only inhibits the level of degradation of ZO-1 and OCLN into the nasal cavity but also enhances the expression of E-cadherin in intercellular junctions. The underlying mechanism might be associated with the nuclear factor erythroid 2-related factor 2/hemeoxygenase-1 (HO-1), a signaling pathway that has been shown to regulate the integrity of the airway epithelial barrier [221, 222]. In addition, a variety of natural products, such as platycodin D, astragaloside IV, glycyrrhizin, etc., have been shown to prevent the development of mucus and the secretion of MUCs in AR [223, 224, 225]. This indicates that these natural substances have a beneficial effect on the AR chemical barrier. The underlying mechanism might involve NF-κB p65. The nuclear translocation of NF-κB has been found to be favorably linked with the expression of MUC5AC [226, 227].

The therapeutic approaches described above primarily target the physical and chemical barriers. Besides, some studies have explored other barriers. The microbiological barrier is one of the areas worth exploring in AR. Probiotic is widely studied in microbiological flora species. In a series of studies, the positive regulatory role of probiotics in intestinal microbiota and AR have been confirmed [228, 229, 230]. Few studies focused on the microbiological barrier in AR patients. In addition, the administration route is one of the important factors that affect the effectiveness of probiotics. Most studies investigated the effects of oral administration of probiotics on AR, whereas few studies investigated the topical/nasal administration of probiotics. The topical application of microbiota was focused on by a recent randomized, double-blind, placebo-controlled, and crossover-involved clinical trial [231]. By intranasal administration, they composed a probiotic combination (PA) consisting of *Lactobacillus rhamnosus* SP1, *Lactobacillus paracasei* 101/37, and *Lactococcus lactis* L1A. Unfortunately, PA did not affect nasal symptoms and peak nasal inspiratory flow in AR patients. In other allergic reactions, the effective effect of nasal probiotics has been confirmed. Nasal administration of the probiotic *Lactobacillus plantarum* forms a probiotic microbiological barrier and induces the production of IFN-γ, IL-12, and IL-10 by immune cells to activate Th1 cells and reduce the increase of EOSs [232]. What is more, it is revealed that nasal probiotic administration reduces inflammatory cells infiltration in BALF in comparison with oral probiotics, which includes a decrease in EOSs, neutrophils, and lymphocytes and a decrease in IL-5 and IgE levels [233]. In vivo experiment revealed that intranasal administration of *L. rhamnosus* GG prevented the development of birch pollen-induced allergic asthma in a strain-specific manner [234]. In the *L. rhamnosus* GG-treated group, it was found that EOSs were significantly reduced in BALF, and the production of pulmonary Th2 cytokines (IL-5 and IL-13) was suppressed as well. This means that, despite the lack of research evidence, the microbiological barrier of the nose is indeed a worthwhile therapeutic entry point to be explored for AR.

### Chronic Rhinosinusitis

Similarly, CRS patients exhibit the compromised epithelial barrier [122, 235]. In many experiments, it is confirmed that CRS is accompanied by impaired TJ and AJ proteins, including decreased CLDN and OLCN, discontinuous CLDN and ZO-1 localization, and decreased JAM-A and E-cadherin [235, 236, 237, 238, 239]. Meanwhile, CRS was accompanied by elevated TEER, sodium uptake, chloride permeability, and ciliary dysfunction [99, 240]. In addition, some CRS patients showed defects in MCC caused by primary cilia dyskinesia [241]. In the exploration of therapeutic measures for CRS, the epithelial barrier is a current entry point of great interest. The currently investigated approaches to restore the epithelial barrier defects in CRS involve protease inhibitors, corticosteroids, microorganisms, etc. Moreover, this chapter will mention some mechanistic advances to restore the epithelial barrier in CRS. The first is the HDAC inhibitor, which is a protease inhibitor and whose positive effect on the AR epithelial barrier has been described above. In NP tissue of CRS patients, the elevated expression of HDAC1 and HDAC9 was observed as well [212]. Its related mechanism may be through the transcription factor p63 mediating TJ disruption. P63 negatively regulates the disruption of TJ. As a member of the p53 family of NF-κB signaling pathway, transcription factor p63 plays an important role in the proliferation and differentiation of epithelium [242]. ΔNp63, a subtype of p63, was reported to be associated with AEC proliferation and E-cadherin [243]. As another isoform of p63, TAp63 is a transcriptional target of NF-κB. In the hTERT-transfected HNECs, it is revealed that knockdown of p63 by small interfering RNA of TAp63 and ΔNp63 induces CLDN-1 and -4 expression and upregulation of SP1 activity, thereby increasing barrier and fence function [244]. Meanwhile, HDAC inhibitors both downregulate p63 and upregulate TJ protein. It suggests that its ability to downregulate p63 may explain the ability of HDAC inhibition to restore epithelial physical barrier defects in CRS. Besides the physical barrier, it is reported that the knockdown of p63 improves the presence of cilia-like structures [244]. Also as addressed in the paragraph on AR, the role of HDAC suggests that HDAC inhibitors are indeed a promising route to repair the physical barrier. HADC inhibitors also prevent IL-4-induced nasal epithelial barrier dysfunction by inhibiting TWIK-related potassium channel-1 [245]. Corticosteroids are used widely in CRS clinics and play a positive role in epithelial barrier defects in CRS. Budesonide significantly increased TEER in CRS patient-derived HNEC monolayers, whose effect is similar to those of hypertonic saline [246].

Finally, taking into account the role of microbiological barriers in CRS, modulation of the microbiological barrier is also an avenue worth exploring. However, this research progress has the same status as in the restoration of microbiological barriers for AR. Few studies focused on the microbiota in nose and the topical/nasal administration pathways. As a class of bacteria that is used in the food industry for many years, *L. lactis* is recognized for its safety. A study composed of nasal and sinus irrigation containing 1.2 × 10^9^ units of live *L. lactis* W136 [247]. Intranasal irrigation of *L. lactis* W163 improved nasal congestion, postnasal drip, and the need to blow the nose in refractory CRS patients. In the 16sRNA microbiome assessment, a lower abundance of *Dolosigranulum pigrum* and no change in α-diversity compared to pre-treatment was indicated. The underlying mechanisms remain to be discovered but may be related to the regulation of the immune response and the bacterial replacement of pathogenic species. It suggests that microbiota containing *L. lactis* W136 can affect the nasal microbiological barrier in CRS patients. In the 16sRNA microbial sequencing results, a lower relative abundance of nasal *Lactobacillus* in patients with CRSwNP and CRSsNP relative to healthy controls is suggested [248]. Furthermore, *Lacticaseibacillus casei* (*L. casei*) is a group of *Lactobacilli* that have been shown to repair defects in the epithelial barrier of the gastrointestinal tract. Ex vitro and in vivo experiments revealed that spray-dried *L. casei* AMBR2 both increased TEER in pNEC of CRSwNP patients in a time-dependent manner and blocked IL-4-induced nasal permeability [248]. In addition, its protection of the physical barrier is reflected in promoting the recombination of the TJ proteins OCLN and ZO-1. In another study, it is found that *L. casei* AMBR2 could not only colonize in the presence of selected donor microbiota but also increase the resistance of the epithelial barrier in the presence of nasal bacteria [249]. Taken together, *Lactobacilli* are worthy of continued exploration in the field of modulating the microbiological barrier for CRS by using local nasal administration. It is unable to ignore the safety issues that create risks when microbiota is applied to the delicate nasal and sinus mucosa directly. In addition to these well-studied therapeutic pathways, there are also less-studied pathways for the repairment of the epithelial barrier. Kappa carrageenan (a sulfated polysaccharide derived from red seaweed) sinus wash was reported to increase TEER and decrease secreted IL-6 levels in the sinus epithelium [250]. Finally, several studies reported potential mechanisms to restore the epithelial barrier in CRS. Apart from the previously mentioned p63, epidermal growth factor (EGF) may also be a promising target. In a study analyzing the microarray datasets, the Gene Expression Omnibus (GEO) database was downloaded to analyze differentially expressed genes in the nasal epithelium of CRSwNP patients and healthy populations [251]. Protein-protein interaction analysis indicated that only EGF was downregulated in NP tissue among the top 10 key differentially expressed genes of CRSwNP. Meanwhile, ZO-1 expression was upregulated after recombinant human (rh) EGF treatment compared with pHNECs of NP tissues. Besides, transwell migration and CCK8 assay showed that rhEGF induced migration and proliferation of HNEpCs. It would suggest that EGF is likely to be another promising target for the treatment of epithelial barrier defects in CRS. Moreover, it is reported that at least 40 gene variants have been associated with disorders of MCC, a component of the chemical barrier [252]. For example, conditional deletion of Dnaic1 in primary cilia dyskinesia mice would result in rhinosinusitis [253]. Unfortunately, there is a shortage of research on treatments focusing on barrier disruption caused by gene variations to treat CRS. This is the research topic that should be expanded upon in the future. In conclusion, repairing epithelial barrier defects in CRS is actually a therapeutic entry point of interest, and some studies are focusing on this, but an increasing number of aspects are still lacking to explore.

## Conclusion

In this review, the compositions of the nasal epithelial barrier, related risk factors, and therapeutic approaches for nasal diseases were described. The nasal epithelial barrier is divided into physical, chemical, immune, and microbiological barriers. They work together to maintain the stability of the nasal mucosa. As one of the main components of the physical barrier, TJs are the functional intercellular complex and are associated with many essential functions of nasal epithelial cells. Among the structural components, CLDNs are integral TJ proteins that form ion-selective pores. OCLN and JAMs play a crucial role in the binding and signaling transduction. In addition, BVES, JAMs, MAGI, MUPP1, and other proteins are also involved in TJ structures. AJs are the important adhesive structure that maintains intercellular connections and avoids structural disruption due to the loosening of cellular contacts. Forming two basic adhesive units, namely, cadherin/catenin and nectin/afadin complexes, they connect the homophilic recognition event to the underlying actin cytoskeleton. Desmosomes which contain the desmosomal cadherins, the armadillo proteins, and plakin proteins cannot be ignored. They maintain intercellular adhesion and cellular integrity. As a channel connecting the cytoplasm of two adjacent cells, gap junctions are essential in the gating of the channel. Mucus is the main component in the chemical barrier. It is the first place where allergens land. It protects the nasal epithelium from drying, preserves the local wetness, and humidifies the inhaled air. When landing in the nasal cavity, inhaled allergens are first trapped by mucus. In the immune barrier, Igs act as immune clearance and inhibit allergic reactions. Defense molecules, including antimicrobial proteins and AMPs, cannot only inhibit inflammation but also promote epithelial repair. Microbiological barriers refer to the microbiota that resides in the surface of nasal mucosa. In addition to regulating barrier homeostasis, permeability, they can also affect TJ proteins. These barriers work together to form the nasal defense against external risk factors and immune response. Meanwhile, under the nasal epithelial barrier hypothesis, this review suggests some risk factors that can damage the epithelial physical, chemical, immune, and microbiological barriers. In the factors, protease-containing allergens, including HDM, pollen, pet dander, insect, etc., occupy a major position. These allergens cleave TJ proteins, increase epithelial permeability, and decrease TER. Some environmental factors cause epithelial barrier disorders as well. PM, DEP, and CS inhibit TJ and AJ proteins, decrease TER, and increase oxidative stress within the nasal mucosa. In addition, the impairment of the epithelial barrier induced by bacteria and viruses has been described. Finally, as evidenced by reduced TJ proteins, the cytokines that are produced in allergic inflammation also exacerbate the damage to the epithelial barrier. Taken together, most of the current studies about the defective epithelial barrier focused on the physical barrier, especially TJs and AJs. Less attention was paid to the remaining parts. The epithelial barrier hypothesis suggests that these triggers should be avoided and that the safety level of exposure to these risk factors needs to be further explored [5].

Given the role of the epithelial barrier in the nose, several studies have been carried out to explore reasonable approaches to address this problem. Several studies on HDAC inhibitors have confirmed that, in AR and CRS, it can restore the nasal epithelial physical and chemical barrier, involving promoting TJ protein expression and reorganizing the TJ network. It means that HDAC inhibitors can correct the defective epithelial barrier function in nasal diseases. However, there are likely to be more completed molecular mechanisms involved in it. One study reported that HDAC inhibitors mediated TJ protein through downregulation of p63. However, it is noted that there are still a lot of unclear mechanistic pathways. For example, genome-wide expression profiles indicate that a few genes are directly affected by HDAC repression [254]. JNJ26481585 (HDAC inhibitor) does not have a monotonic effect on epithelial barrier function and is less effective at higher doses [214]. As they are commonly used clinically in AR and CRS, corticosteroids have also been studied and confirmed to have a positive effect on the physical barrier. Also, natural products isolated from plants also have a protective and repairing effect on the nasal epithelial barrier. Finally, there are also therapeutic approaches targeting the microbiological barrier. The microbiome is manipulated to influence the nasal microbiological barrier for AR and CRS. Probiotics are a beneficial flora that has been studied extensively. Increasing evidence suggests that nasal administration of probiotics can have a protective effect against AR. Through the formation of a probiotic barrier in the nasal epithelium, the body's ability to resist Th2 responses can be strengthened. Moreover, *Lactobacilli* are worth exploring in both AR and CRS. Evidence suggests that there is a low relative abundance of *Lactobacillus* in CRS. By repairing the physical barrier and manipulating the microbiological barrier, the topical administration of *Lactobacilli* to the nasal cavity has been shown to restore the barrier through indirect and direct studies. All the evidence suggests that restoring the epithelial barrier may be a promising therapeutic route. However, the research is still in its infancy, and there are certain difficulties. Most studies of epithelial barrier (particularly the microbiological barrier) are characterized by limited study cohorts and variable test methods. In addition, there is a lack of experimental models with commonality in the explorations of the epithelial barrier. For example, the universally applicable animal models are required for the study of researching the nasal microbiological barrier. This hinders the monitoring of the impact of risk factors on the barrier and the development of new therapeutics. In addition, the ideal dose and timing have not been addressed in any of the current investigations of these innovative methods. It is also important to consider their adverse effects.

In summary, the epithelial barrier is associated with nasal allergic diseases. This review describes their compositions and risk factors. Also, some novel approaches to target the epithelial barrier to treat AR and CRS are also presented. Although there are still some deficiencies and challenges to overcome in the future, restoring the epithelial barrier may be a promising strategy for the development of nasal disease therapies.

## Conflict of Interest Statement

The authors have no conflicts of interest to declare.

## Funding Sources

This work was supported by Chengdu University of Traditional Chinese Medicine Graduate Student Research and Innovation Program (grant number, CXZD2021003).

## Author Contributions

Rong Zhang and Lan Zhang contributed to the conception of the review article and to draft and revise the article. Peishan Li, Kaiyun Pang, and Huixia Liu contributed to figure drawing. Li Tian supervised and reviewed the manuscript. All the authors approved the final version of the article. The accuracy of any part of the article was appropriately investigated and resolved in agreement with all the authors.

## Notes

Edited by: H.-U. Simon, Bern.
